# Clinical and perceived quality of care for maternal, neonatal and antenatal care in Kenya and Namibia: the service provision assessment

**DOI:** 10.1186/s12978-016-0208-y

**Published:** 2016-08-11

**Authors:** Nadia Diamond-Smith, May Sudhinaraset, Dominic Montagu

**Affiliations:** Global Health Group, University of California San Francisco, San Francisco, CA USA

**Keywords:** Quality of care, Delivery, Maternal health, Sub-Saharan Africa, Large nationally representative datasets

## Abstract

**Background:**

The majority of women in sub-Saharan Africa now deliver in a facility, however, little is known about the quality of services for maternal and newborn basic and emergency care, nor how this is associated with patient’s perception of their experiences.

**Methods:**

Using data from the Service Provision Assessment (SPA) survey from Kenya 2010 and Namibia 2009, we explore whether facilities have the necessary signal functions for providing emergency and basic maternal (EmOC) and newborn care (EmNC), and antenatal care (ANC) using descriptives and multivariate regression. We explore differences by type of facility (hospital, center or other) and by private and public facilities. Finally, we see if patient satisfaction (taken from exit surveys at antenatal care) is associated with the quality of services (specific services provided).

**Results:**

We find that most facilities do not have all of the signal functions, with 46 and 27 % in Kenya and 18 and 5 % in Namibia of facilities have high/basic scores in routine and emergency obstetric care, respectively. We found that hospitals preform better than centers in general and few differences emerged between public and private facilities. Patient perceptions were not consistently associated with services provided; however, patients had fewer complaints in private compared to public facilities in Kenya (−0.46 fewer complaints in private) and smaller facilities compared to larger in Namibia (−0.26 fewer complaints in smaller facilities). Service quality itself (measured in scores), however, was only significantly better in Kenya for EmOC and EmNC.

**Conclusions:**

This analysis sheds light on the inadequate levels of care for saving maternal and newborn lives in most facilities in two countries of Africa. It also highlights the disconnect between patients’ perceptions and clinical quality of services. More effort is needed to ensure that high quality supply of services is present to meet growing demand as an increasing number of women deliver in facilities.

## Background

An increasing number of women in Sub-Saharan Africa are seeking care for delivery and antenatal care (ANC) services in health facilities [1]. In Kenya and Namibia, the countries of focus in this analysis, facility deliveries are increasing, with the number of births in the last 5 years in a health facility rising to 61.2 % in the 2014 Kenyan DHS (up from 43 % in 2008–09) and 87.4 % in the 2013 Namibian DHS (up from 81 % in 2006–07) [2, 3]. Governments and the international community have supported this trend in the hopes that delivering in facilities would give women access to higher quality care and reduce adverse maternal and neonatal outcomes. However, recent data in some countries has suggested that the quality of services available in many health facilities might be so poor as to provide few health benefits. In Low- and Middle-Income Countries growing evidence suggests that despite high levels of deliveries in facilities, maternal and neonatal mortality remains stubbornly high. For example, in India, increasing numbers of women delivering in facilities has not led to any decrease in maternal and neonatal mortality [4]. As a result of this, experts are realizing the importance of exploring the context and content of care in a holistic way, from understanding the quality of services offered at facilities through to patients perceptions of that care [5]. A few studies have begun addressing this issue by looking in depth at what quality of care indicators for maternal and neonatal emergency care are actually available at facilities in low income countries. For example, Nesbitt et al. [6] found in a survey of facilities that provide delivery in Ghana that only 18 % of births were occurring in facilities with high levels of care. Kyei et al. [7] explored the quality of antenatal care in Zambia using two datasets, and found that only 3 % of facilities had optimum ANC care, and almost half provided less-than-adequate services. A study of newborn care in hospitals in Kenya found that hospitals generally had only 30 to 56 % of items necessary to care for sick newborns [8].

As more and more women deliver in facilities, it is important to understand what type of essential services are actually available to women so that we are better able to target gaps and improve the overall quality of delivery care and ANC.

Quality for maternal and neonatal health (MNH) has both clinical and non-clinical components, each measured in terms of structure (inputs), processes (actions), and outcomes (results) [9]. Structural quality is the most straightforward to measure – being largely static and observable at any time. Process quality is much harder, being specific to cases, providers, their interaction, and the presentation and response to illnesses, often rare and difficult to observe. Outcomes are likewise hard to observe due to temporal delays and challenges of attribution to a single therapy or the quality of inputs and actions. As a result, most data and most analysis focuses on structural quality.

Service Provision Assessment (SPA) surveys provide high quality data that has been used by many researchers studying health related outcomes. There is a plethora of data in the SPA surveys, which have been used for various purposes, including calculating USAID and WHO’s service readiness indictors. However, thus far SPA data has not been used extensively in the published literature, in part because linking SPA and DHS data is quite difficult as the two surveys are not collected in the same years or in the same facilities within each country (although they are both representative and usually cover the same regions).

Clinical quality of care measures for maternal health have been widely discussed in the literature, focus primarily on safety and effectiveness, and are constantly evolving. Gabrysch et al. [10] argued for an expansion of the traditional focus on Emergency Obstetric Care (EmOC) indictors to include routine maternal care and newborn emergency (EmNC) and routine care. Nesbitt and colleagues used these recommended and expanded set of signal functions to look at facility quality in 86 facilities in Ghana [6]. In this analysis we study the same signal functions (routine, EmNC, and EmOC) used by Nesbitt et al. [6] to look at indicators of care in two other African countries, Kenya and Namibia. We also expand their focus to include indicators of quality for antenatal care (ANC), and then add an examination of patient experience.

The WHO Quality of Care Framework outlines various components of quality of care for maternal and newborn health, a few of which fall under the heading of patient experiences [11]. The patient experience quality domain has been little studied despite evidence of its importance. Research has shown that women’s decisions about where they will go for care is influenced by the types of interactions they have with providers in the past, and their perceptions of quality of care provided in the facility. A cross-sectional study in Kenya found that women who had lower perceived quality of ANC services were more likely to have had their first ANC visit late [12]. Perceived quality during ANC visits influences women’s decision about delivering in a facility at all [13]. Past research in Tanzania explored both the patient’s experience of care and clinical measures of quality, but did not analyze associations between these two domains [14]. Another study in Kenya found little associations between women’s reports of their care and observed indicators of care received during labor and delivery [15].

This analysis compares indictors of antenatal, maternal, and neonatal emergency care collected in Kenya in 2010 and in Namibia in 2009. We compare the distribution of services provided by hospitals, health centers and other facilities, in both private and public facilities. We also explore the correlation between respondents’ reports of complaints experienced in their ANC visits (from exit interviews), the services they received, and the type and ownership of facility they attended.

The specific objectives of this paper are to: 1) Describe the clinical quality of facilities in Kenya and Namibia; 2) Describe the perceived quality of patients exiting facilities; and 3) Determine if perceived quality is predictive of clinical quality.

## Methods

Service Provision Assessment (SPA) surveys are nested within select Demographic and Health Surveys (DHS) that have been conducted in a number of countries in Africa. The DHS, and SPA are cross-sectional, nationally-representative surveys. They use model questionnaires, which can be adapted by each country. Manuals and technical assistance ensure similar procedures are followed across countries. Detailed reports on each national DHS and SPA, describing details of sampling, fieldwork, and characteristics of respondents can be obtained from the DHS website (measuredhs.com). Datasets are available in a public repository accessible from the same website. SPA surveys collect information about different facility-based health services and how ready facilities are to provide those services. It includes topics relating to maternal, newborn and child health, infrastructure, resources and systems, and other health issues. The goal is to provide a comprehensive view of a country’s formal health care system. Given that this analysis involves secondary analysis of publically available, de-identified data, we did not seek ethical approval.

This study uses SPA data from Kenya (2010) and Namibia (2009), chosen because they are two Sub-Saharan African SPA surveys collected at roughly similar time points. From an initial sampling frame of all facilities in a country, facilities are categorized by type and managing authority (private/public). Hospitals are generally oversampled, and 400 to 700 facilities are surveyed using five different modules: service readiness indicators, exit interviews, health worker interviews, direct observations and inventory (medications available, etc.). Data from the service readiness indicators and exit interview modules are used in this analysis. While other surveys exist that measure the clinical quality of care and service readiness of maternal and neonatal health, such as WHO’s Service Availability Readiness Assessment, we choose to use the SPA data because it has the added exit interview component and is therefore able to compare patient experiences with clinical measures.

### Delivery service readiness indicators

Data from three parts of the service readiness indictors was used in this analysis: antenatal care (ANC), delivery care and neonatal care. We calculated the percent of each facility-type reporting provision of delivery or ANC services, by country. We show the breakdown of the various indictors by facility type and level. Each country classified facility levels slightly differently, however both had a “hospital” indicator. We categorized only the first level health center listed as “health center.” All other facilities were grouped into “other facility.” For the classification of ownership (public vs. private) of facility both countries had a category for “public or government”, and everything else (private/NGO/mission/faith based) was grouped into “private.”

The quality indicators used in this analysis are based on Nesbitt et al.’s [6] quality index dimensions for maternal and neonatal care. This includes indicators for quality of routine delivery care, emergency obstetric care (EmOC), and emergency newborn care (EmNC). Nesbitt also proposed a fourth dimension, non-medical quality, measured through questions about availability and cleanliness of a toilet with water and soap for hand washing, and if a woman can have a delivery companion. This was not included in this analysis because the majority of these indicators were not collected in any of the SPA surveys (Table 1) [6].

**Table 1:** Routine delivery, EmOC and EmNC quality indicators [6]

Indicator	Indicator in SPA	Kenya (%)	Namibia (%)	Comments
Routine Delivery Care				
Monitor Labor with Partograph	Partograph (observed)	75.93	50.00	
Infection prevention	Observed hand disinfectant	35.24	55.08	
Measure Blood Pressure	Take BP, routine	71.10	43.36	
Controlled cord traction	Apply cord traction, routine	89.08	63.28	
Injection of Oxytocin within 1 minute of delivery	Injectable oxytocin/syntocin, observed, valid date	79.40	45.31	
Uterine massage	Massage fundus through abdomen, routine	88.09	83.98	
Place baby on abdomen	Delivery of baby to abdomen	49.13		Only Kenya
Dry baby immediately	Dry and wrap newborns to keep warm	98.26		Only Kenya
Apply eye ointment	Apply tetracycline eye ointment to both eyes	81.14	65.23	Only in Kenya, in other countries used “Antibiotic eye drops/ointment (not chloramphenicol)”
Weigh baby after delivery	Two indicators: “Weigh baby, routine” & “newborn scale observed”	98.76	89.84	Used “newborn scale observed” as indictor
Initiate Breastfeeding within 1 hour	Breastfeeding 1st hour	95.29		Only in Kenya
Delay bathing at least 6 hours after delivery	Give full bath within minutes/few hours after	90.57	41.41	Only in Kenya, in Namibia “gave bath within 24 hours”, removed from analysis because confusing
Emergency Obstetric Care (EmOC)				
*Basic functions*				
Parenteral Antibiotics	Ever or past 3 months: used parenteral antibiotics	68.73	16.80	
Parenteral Oxytocin	Injectable oxytocin/syntocin, observed	79.40	45.31	
Parenteral anti-convulsing	Injectable diazepam observed, /Injectable magnesium sulfate observed	74.69 / 52.36	68.75 / 14.84	
Manual removal of placenta	Used manual placental removal, past 3 months	48.64	11.33	
Manual removal of retained products of conception	Past 3 months: retained products extracted	70.43	37.50	
Instrument delivery	C-sect: sterile instruments in tray/drum package	93.33	86.11	
*Comprehensive*				
Blood transfusion	Provide blood transfusion	25.06	10.55	Also looked at ability to transfer for blood transfusion
C-section	Use indictors for “instrument delivery” above			
Emergency Newborn (EmNC)				
*Basic Functions*				
Injectable antibiotic for newborn sepsis	Injectable amoxicillin/ampicillin observed, Injectable gentamicin observed	9.18 / 55.09	16.80 / 28.52	
Newborn resuscitation with mask and bag	Infant resuscitation bag/mask or tube/mask) observed	81.64	62.11	
Kangaroo mother care	Kangaroo mother care, routine	50.37		Only in Kenya
Express milk with spoon	NA			
Dexamethasone	NA			
*Comprehensive*				
IV fluids for newborn	IV fluid Observed /IV infusion set observed	88.56 / 91.32	86.33/ 96.88	
Antenatal Care (ANC)				
Guidelines on ANC	NA			
Staff trained in ANC	Protocols: teaching aids for ANC, observed	58.72	36.50	
Blood pressure apparatus	Take Blood pressure, routine, observed	68.75	38.20	
Hemoglobin	Blood test for anemia, routine, observed	39.51	30.41	
Urine dipstick- protein	Urine test for protein, routine, observed	36.47	33.09	
Iron tablets	Iron tablet available, observed	45.18		Only in Kenya
Folic Acid Tablets	NA			
Tetanus toxoid Vaccines	Available today	95.86	94.24	
Other quality indicators				
Number of days a week ANC offered	ANC offered 5+ days a week	84.58	42.09	
Treat STIs	Routinely treat STI	59.17	68.13	

*NA = not available in any surveys

The ANC quality indictors are comprised of the data identified by the DHS as tracer indicators of ANC quality, together with two more variables for whether the facility treats STIs at ANC visits and if ANC is offered at least 5 days a week (Table 1) [16]. We assumed that if a facility indicated that it provided a specific service (routine delivery care, EmOC, EmNC or ANC), it should have all of the recommended quality components, regardless of facility type (hospital, health center, other facility).

For many of the indictors the response options included both “reported” and “observed.” For all of the indictors that had the option of “observed” we only used that response to make this the most conservative estimate. As can be seen in Table 1, not all indicators were available in both countries, with the most recent survey (Kenya, 2010) having the most complete set of indicators. Additionally, some questions were asked slightly differently in different surveys. Table 1 shows the indicators suggested by Nesbitt et al. [6], the variable collected in the SPA that we used to measure that indicator, and whether and how it differed by country . Information about the practices of bathing newborns was asked in Kenya as “Do you give a full bath within minutes/few hours after birth”, however, in Namibia it was asked as “Do you give a full bath within 24 h.” Past evidence has suggested that in this setting bathing too soon is associated with increased risk of neonatal mortality, however, bathing the infant after a few hours is recommended [17, 18]. The two different wordings of this question make it difficult to determine if the newborn was bathed at the recommended time and so this indictor was dropped from this analysis.

### Exit interview module

Data from exit interviews with clients of antenatal centers included questions about the types of services they received, the complaints they reported, how long they had to wait and pay, and whether this facility was the closest facility to their home. Clients were asked a series of questions about whether they had any complaints about their experience with ANC (Table 2). These were combined into a “complaint” score. Unfortunately, there are no exit interviews with women after giving birth and so we were not able to include information on women’s perceived problems after delivery. We made an average complaint score for each facility equal to the average of the number of complaints listed by each client exiting that specific facility after an ANC visit.

**Table 2:** Percent of ANC clients that reported each type of complaint by country

Complaint Indicators	Kenya (N = 1445)	Namibia (N = 880)
Time had to wait	57.78 %	40.11 %
Ability to discuss problems/concerns	8.30 %	8.64 %
Amount of explanation for problem/treatment	8.37 %	9.09 %
Quality of exam	5.33 %	5.34 %
Visual privacy	4.98 %	2.39 %
Auditory Privacy	4.98 %	2.50 %
Availability of medicines	19.10 %	6.93 %
Hours of service	13.56 %	8.98 %
Days of service	8.17 %	9.43 %
Staff treatment of client	7.68 %	12.61 %
Cost	6.44 %	7.84 %
Cleanliness of facility	16.19 %	2.05 %

### Scores

We constructed a score for facilities based on Nesbitt et al. [6] categorization, including substandard, low, intermediate and high/basic for the routine delivery care, EmNC, and EmOC indicators. In their analysis, routine delivery had a maximum of 12 indicators, EmNC had 6 and EmOC had 8. Not all of these same indictors were collected in the SPA dataset in both Namibia and Kenya, unfortunately. A total of 8 routine delivery indictors, 3 EmNC and 6 EmOC were available in both countries. Since making a score from only 3 indicators would not give nuanced information, we did not make an overall score of EmNC indicators. Since fewer indicators were available for each of the composite scores, we adjusted the cutoff for the score rating (substandard, low, intermediate and high/basic) to be one indicator lower than was used by Nesbitt et al. [6].

We measured patients’ perceived quality of care by the number of complaints that they reported from their ANC visit. We then created a facility level complaint score, which was the average of the scores of all of the clients interviewed exiting that facility. This was the main outcome of interest in the analysis.

### Statistical analysis

All analyses were performed using Stata 13MP. First, we tested whether service-level measures of ANC quality collected in the service readiness questionnaire were associated with a patient’s perceived quality of their ANC visit, as measured in the exit interviews. Descriptive analyses were performed by country across routine delivery, EmOC and EmNC quality indicators.

We conducted multivariable linear regressions in order to test associations between facility quality and patients’ perceived quality as measured by the complaint score. Analyses were stratified by country and clustered at the facility level. The multivariable regressions included a number of facility quality and service related factors that could be associated with patients’ perceived quality. Three indicators were included to measure the quality of information or provider interaction (i.e. the percent of clients per facility that reported that the provider asked them if they had any questions while they were at their visit, if facility offered ANC 5 or more days a week and if the facility had training materials for ANC (observed)). The multivariable regressions also included five service indictors of quality: if tetanus was available that day, urine tests, blood tests, blood pressure, and if the facility treats STIs. Availability of iron tablets was also included in the Kenya model. We also included categorical variables for the type of facility (hospital vs. health center vs. other) and ownership of facility (private vs. public).

Finally, we conducted two multivariable linear regressions (one for each country) looking at the association between type of facility (hospital vs. health center vs. other) and ownership of facility (private vs. public) and quality scores created by summing the number of indicators in each category (Routine, EmOC, EmNC and ANC), shown in Table 1.

## Results

The number of hospitals, health centers and other health facilities differed greatly between countries. In Kenya, 59 % of the sampled facilities that provided deliveries were hospitals, 20 % were health centers and 21 % were other types of facilities. Of those sampled that offered ANC, 43 % were hospitals, 18 % were health centers and 40 % were others. The majority of facilities sampled that offered deliveries or ANC were public. In Namibia, 17 % of the facilities offering deliveries were hospitals, 15 % were health centers, and 68 % were other facilities. Of facilities that offered ANC, only 3 % were hospitals, 14 % health centers and 83 % others. The vast majority of facilities sampled for both deliveries and ANC were public. Both countries had more public facilities included in the sample than private facilities.

### Distribution of selected routine, EmOC, EmNC and ANC indicators

**Fig. 1:**
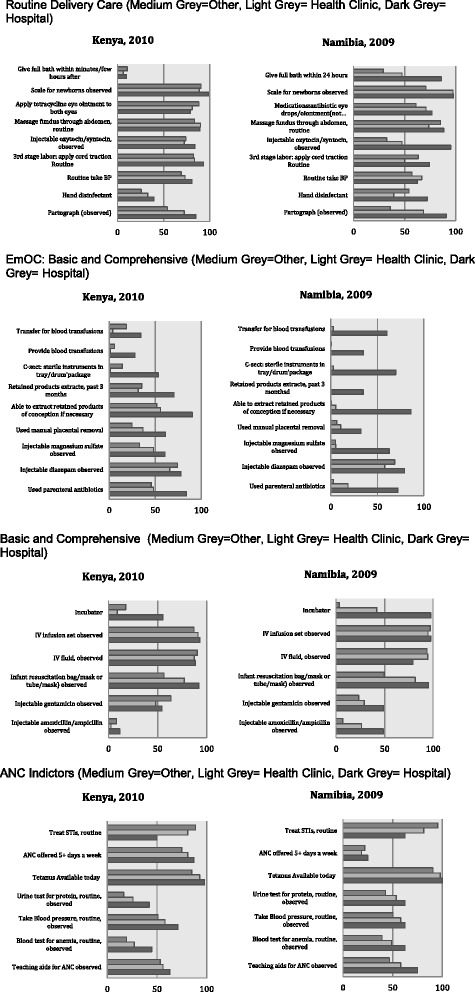
Routine Delivery Care, EmOC: Basic and Comprehensive, EmNC: Basic and Comprehensive, and ANC Indictors (Medium Grey = Other, Light Grey = Health Center, Dark Grey = Hospital)

Quality of care differed by both facility level/type and across countries based on routine delivery, EmOC, EmNC and ANC indicators. Below we discuss each indictor by facility type and country. The information is shown graphically in the graphs in Fig. 1.

### Routine delivery

Overall, Kenya performed better than Namibia in the majority of routine delivery indicators. Scales for newborns were available in virtually all hospitals in both countries, and over 80 % of other facilities in Kenya and health centers in Namibia. Scales were only available for about 70 % of “other” facilities in Namibia. Over 80 % of all facilities applied tetracycline eye ointment to both eyes in Kenya, and between 60 and 80 % of all facilities had antibiotic eye drops in Namibia. While injectable oxytocin was observed in over 75 % of facilities in Kenya, it was observed in less than 50 % of non-hospital facilities in Namibia, although over 90 % of hospitals had it. Routine application of cord traction in the 3^rd^ stage of labor occurred in over 80 % of all facilities in Kenya, but only about 60 % in Namibia. Blood pressure was routinely taken in between 70 and 85 % of facilities in Kenya, but only about 60 % in Namibia. A partograph was observed in over 80 % of hospitals in both countries, about 70 % of health centers, but less than 50 % of other facilities. Almost all facilities of all types dried and wrapped the newborn to keep warm in Kenya, although only about 50 % of facilities delivered the newborn to the abdomen routinely (these questions were not asked in Namibia).

### EmOC

Facilities performed more poorly on indicators of emergency obstetric care, with the exception of breastfeeding, which was only asked in Kenya, and which all facilities preformed very well (almost 100 %). Just under 40 % of hospital provided blood transfusions in both countries, and very few other facilities did. While about 60 % of hospitals in Namibia and over 50 % of hospitals in Kenya reported that they transferred for blood transfusion, very few other facilities or health centers did. Sterile C-section instruments were observed in about 70 % of hospitals in Namibia and 50 % in Kenya, and very low rates of these were observed in other facilities. Retained placenta products had been extracted in the last 3 months in less than 40 % of hospitals in Namibia and not at all in other facilities, and in Kenya, this had be done in 70 % of hospitals and about 35 % of other facilities. Similarly, the majority of hospitals (>80 %) in Namibia had the ability to extract retained products of conception, and almost no other facilities in Namibia did, whereas over 80 % of hospitals and about 50 % of other facilities and health centers had this ability in Kenya. Injectable magnesium sulfate was observed in over 60 % of hospitals in Namibia and Kenya, almost 50 % of health centers and about 30 % of other facilities in Kenya, and virtually no non-hospital facilities in Namibia. Injectable diazepam was observed in 60 % or more facilities in both countries (with Kenya having higher rates over all) (injectable magnesium sulphate and injectable diazepam, the former being recommended WHO treatment, and the latter an alternative still included in national guidelines). Finally, parenteral antibiotics had been used in over 80 % and 70 % of hospitals in Kenya and Namibia, respectively, about 50 % of other types of facilities in Kenya, and less than 20 % of other facilities in Namibia.

### EmNC

Almost all hospitals in Namibia had an incubator, whereas only about 50 % in Kenya did. Over 40 % of health centers in Namibia had an incubator, and less than 20 % of other facilities in Namibia and health centers in Kenya had an incubator. IV infusion sets and fluid were observed in over 80 % of all facilities in both countries. Over 80 % of hospitals in both countries had an infant resuscitation bag/mask, along with almost 80 % of health centers, and over 50 % of other facilities. Injectable gentamicin was observed in over 50 % of all facilities in Kenya, and about 50 % of hospitals and less than 30 % of other facilities in Namibia. Finally, injectable amoxicillin/ampicillin was observed in very few (about 10 % or less) of all facilities in Kenya, but about 50 % in hospitals in Namibia, almost 30 % of health facilities, and 10 % of other facilities. A question about kangaroo mother care was only asked in Kenya, and about 50 % of all facilities reported doing this routinely.

### ANC

The majority of facilities routinely treated STIs in both countries, with over 90 % of other facilities, 80 % of health centers and 50 % of hospitals providing this service. ANC was offered more than 5 days a week at over 70 % of all facilities in Kenya, however, in only about 20 % of all facilities in Namibia. Tetanus shots were available daily at over 80 % of all facilities. Urine tests were observed and routinely provided in over 60 % of hospitals in Namibia and 40 % in Kenya, over 50 % of health centers in Namibia and about 30 % in Kenya, and 40 % of other facilities in Namibia and under 20 % in Kenya. Blood pressure was observed and routinely taken in over 50 % of all facilities in both countries. Anemia was routinely tested in between 40 and 60 % of all facilities in Namibia, but only 20–50 % of all facilities in Kenya (with hospitals preforming the best in both countries). Teaching aids for ANC were observed in between 50 and 60 % of facilities in Kenya, and slightly more so in hospitals in Namibia. Data about the availability of iron tablets was only collected in Kenya, and there, iron tablets were observed in about 60 % of other centers, and 50 % of hospitals and health centers.

Differences between private and public facilities were small in terms of indictors, with public facilities preforming better on some indictors and private on others (Data not shown). Private facilities had higher quality of EmNC services in general, and public facilities preformed better in terms of the ANC indictors, in general (with exceptions in both cases).

### Routine delivery and emergency obstetric care scores

**Fig. 2:**
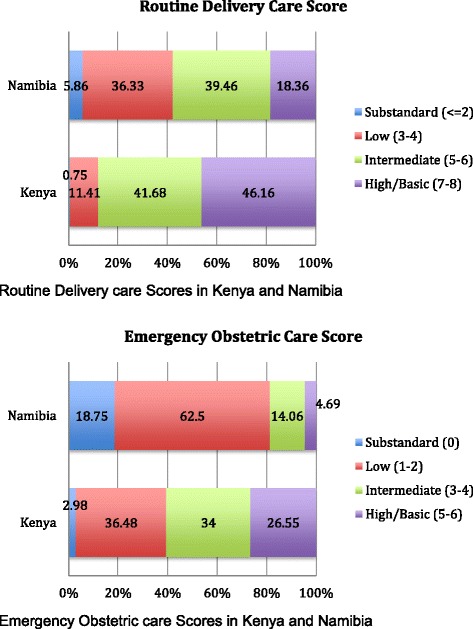
Routine Delivery and Emergency Obstetric care scores in Kenya and Namibia

As can be seen in Fig. 2, about 5 % of facilities in Namibia scored “substandard” in their score of routine delivery care, about 35 % got a score of “low”, over 36 % a score of intermediate, and 18 % a score of high. Kenya scored better overall, with less than 1 % have a substandard score, almost 12 % a score of low, 42 % a score of intermediate, and 46 % a high score.

In terms of emergency obstetric care, almost 20 % of facilities in Namibia were substandard, 62 % had a low score, 14 % a intermediate score, and less than 5 % a high score. Kenya again performed better, with about 6 % of facilities substandard, 36 % receiving a low score, almost 40 % an intermediate score, and 18 % a high score (Fig. 2).

### Perceived quality

More complaints were reported in Kenya than in Namibia (Table 2). The most commonly cited complaint was wait time, where 57.8 % of women in Kenya and 40.1 % of women in Namibia reported that the time they had to wait for their ANC visit was a problem. The next most commonly reported complaint in Kenya was availability of medications (19.1 %), followed by cleanliness of the facility (16.2 %), and hours of service (13.6 %). The remaining complaints were cited by less than 10 % of respondents. In Namibia, the second most commonly cited complaint was the staff treatment of the client (12.6 %), and although no other complaints were reported by over 10 % of respondents, 9.4 % reported the days of services provided were a problem, and 9.1 % reported that the amount of explanation for their problem/treatment was lacking.

#### Multivariate regression: factors associated with high complaint score

The main outcome of the first analysis was the average facility level complaint score. A higher score means that more complaints were listed on average by exiting ANC clients. Clients exiting private ANC facilities gave statistically significantly lower complaint scores compared to those exiting public facilities by −0.46 (*p* < 0.01) in Kenya and −0.25 (not statistically significant) in Namibia (Table 3). Receiving ANC in a higher-level facility (compared to lower level) was associated significantly with more complaints in Namibia (*p* < 0.05) and was in the same direction, but not significant in Kenya.

**Table 3:** Regression of predictors of average facility level complaint score reported by patients exiting ANC centers, by country, Coefficient [Robust standard error]

	Kenya	Namibia
*N*	1445	880
Private compared to public facility	−0.46 [−5.29]***	−0.25 [0.17]
Facility Category (higher is smaller)	0.03 [0.50]	0.26 [0.12]**
Percent asked if patient had questions	0.10 [0.98]	−0.38 [0.17]**
ANC offered 5+ days a week	0.04 [0.41]	0.25 [0.31]**
Has training materials for ANC	−0.00 [−0.06]	−0.31 [0.20]
Tetanus available today	0.10 [0.45]	0.09 [0.07]
Offers Urine tests, routine, observed	−0.01 [−0.08]	−0.17 [0.28]
Iron	−0.15 [−1.90]*	
Blood test for anemia (Hemoglobin), routine, observed	0.15 [0.96]	0.45 [0.21]**
Offers Blood Pressure, routine, observed	0.29 [3.07]***	−0.22 [0.30]
Treats STIs	0.26 [3.30]***	0.01 [0.24]
Constant	1.39 [4.56]***	1.03 [0.44]**

*< 0.1, **< 0.05, ***< 0.01

In Namibia, being asked by a provider if they had questions was associated with clients reporting fewer overall complaints. ANC being offered more than 5 days a week was associated significantly more complaints in Namibia and was not significant in Kenya.

In terms of services offered, no real trend emerged for any indicators. Hemoglobin tests were significantly associated with a higher complaint score in Namibia, and not significant in Kenya. Measuring blood pressure routinely was significantly associated with a higher complaint score in Kenya, and not significantly associated in Namibia. Finally, treating STIs was significantly associated with a higher complaint score in Kenya, and not significantly associated in Namibia. Offering iron (data on this was only collected in Kenya) was marginally statistically significantly (*p* < 0.1) associated with fewer complaints in Kenya. The other indicators were not significantly associated in either country.

#### Multivariate regression: factors associated with higher care scores

Overall, only small differences emerged between private and public facilities for each indicators (data not shown). Thus, we created a score for each area of expertise (Routine Care, EmOC, EmNC and ANC) based on the data available for each country shown in Table 1. We then regressed facility type (hospital, health center, other facility) and private versus public, on each of these score separately by country (Table 4). Private facilities scored significantly higher in EmOC and EmNC scores in Kenya, and marginally (*p* < 0.1) higher for EmOC in Namibia. Being in a lower level facility was associated with lower scores for every topic, compared to hospitals, with the exception of ANC for Namibia, where lower level facilities had higher scores. “Other” types of facilities were associated with lower scores on all indictors than health centers.

**Table 4:** Regression of relationship between type of facility (public/private), level of facility (hospital, health center, other) and score of routine care indicators

	Routine Care Score	EmOC Score	EmNC Score	ANC Score
KENYA (*N* = 1744)				
Private (compared to public)	0.006 [0.14]	0.51 [0.6]***	0.12 [−0.05]***	−0.08 [0.09]
Facility Type (compared to hospital)				
Health center	−1.57 [0.17]***	−1.92 [0.08]***	−0.74 [0.08]***	−0.16 [0.12]
Other	−5.62 [0.13]***	−2.90 [0.07]***	−2.51 [0.07]***	−1.38 [0.10]***
Namibia (*N* = 1111)				
Private (compared to public)	−0.43 [0.17]	0.09 [0.05]*	−0.04 [0.10]	−1.32 [0.14]
Facility Type (compared to hospital)				
Health center	−2.15 [0.28]***	−2.77 [0.08]***	−0.26 [0.17]	0.78 [0.23]***
Other	−3.80 [0.25]***	−3.06 [0.07]***	−1.55 [0.15]***	0.78 [0.20]***

*< 0.1, **< 0.05, ***< 0.01

## Discussion

In this study we assess the quality of antenatal, maternal and neonatal emergency care in Kenya and Namibia using large, nationally representative data. Furthermore, we are able to test for relationship between facility level quality indicators and patients experiences of quality for ANC services. In both countries we found that the majority of facilities where women are delivering are not prepared to provide even medium levels of emergency obstetric care. In Namibia 82 % of facilities were categorized as substandard-to-intermediate; Kenya did slightly better, but the majority (54 %) of facilities were still categorized as substandard-to-intermediate. Our findings indicate that facilities fare even worse for emergency obstetric care: about 95 % in Namibia and 75 % in Kenya were categorized as substandard-to-intermediate. As mentioned above, 87 % of women in Namibia and 61 % of women in Kenya now deliver in a health facility [2, 3]. This indicates that an increasing number of women are exposed to substandard care, which could lead Kenya and Namibia to experience similar experiences as other countries, such as India, that dramatically increased the number of women delivering in facilities without the necessary improvement in quality of care.

### Public-private

Despite many indictors being fairly similar in public and private facilities, there were some important differences in routine, EmOC, EmNC and ANC. Overall, there were no differences between private and public facilities in either country for Routine care or ANC, and private facilities preformed better in Kenya on EmOC and EmNC (no significant differences in Namibia). There are many possible explanations for the differences in private compared to public facilities, including more resources (perhaps due to patient fees or charitable funding sources), better trained or incentivized staff, higher quality standards or expectations, etc.

### Patient perceptions

Patients’ perceptions of quality were not consistently associated with any of the clinical measures of quality that we explored. Private facilities had lower patient-reported complaint scores in Kenya, despite ANC quality generally being lower in private facilities (although other types of quality were higher in general in private facilities). Past research in Tanzania that found few differences between public and private facilities in ANC care also found that patients assess the quality of private facilities higher than public ones [14]. In Namibia receiving treatment at a lower level facility was associated with fewer complaints, but this did not hold for Kenya. It is possible that at lower level facilities staff are less rushed and have more time to spend with clients, and therefore provide more patient-centered, personal, interactions, and allow for shorter wait times. However, it is important to note that despite people’s perceptions of quality being better at private facilities, there was no significant association between type of facility (private/public) and the ANC quality score.

Patients in Namibia reported fewer complaints in facilities where, after interactions, providers asked questions of them and ANC services were offered more frequently. This supports past literature that found that factors influencing perceived quality revolve primarily around patient-provider interactions [19, 20]. Patient perceptions were not consistently associated with measures of the clinical services provided by facilities, such as treating STIs, taking blood pressure, etc. There has been recent evidence that patient perceptions of good quality are highly associated with proper medical care in outpatient settings however our findings suggests that perceived and actual quality measures may not be strongly associated for more complex inpatient services [21]. It is possible that these measures are not appropriate indicators of the types of clinical services that impact patient perceptions, or that other factors, such as provider-patient interactions are actually more important factors influencing patient perceptions. Past research in Kenya that compared women’s reports of care received and observations of care found large disparities, and suggested that factors such as whether the woman had a cesarean section impacted her perception of what services and care she received [15].

There is increasing evidence around the world, of mistreatment experienced by women during childbirth, and other reproductive health services [22]. Research in Kenya found that 20 % of women reported disrespect and abuse while seeking maternal health care services, including lack of privacy, disrespect, neglect and abandonment, lack of consent, physical abuse, and requests for bribes [23]. Our analysis adds to this body of research highlighting the magnitude of importance of poor quality of care and the impact that may have on services utilization.

### Limitations

There were a number of limitations to the study. First, it is difficult to compare across countries and surveys without more systems-level data, including government health expenditures, underlying chronic disease conditions, environmental factors, and literacy levels. Macro-level data would help ground these findings in context. There was one year of time difference between the Kenya and Namibia surveys, so part of the reason that Namibia lagged slightly could be due to the fact that the data was collected earlier. The types of facilities that fell into the categories of ownership (public/private) and level in the two countries differed, and there could have been different patient or administrative expectations about what services should be provided at each facility level and type. In both Kenya and Namibia some NGOs and faith based health facilities are supported by the government, and these facilities could have been misclassified as “private.”

One of the main limitations of the Namibia sample is that many fewer hospitals and health facilities were included in the SPA dataset compared to “other” facilities. We have little knowledge of what these other types of facilities really are. We are limited in both our certainty about the findings from hospitals and health facilities (since the samples are smaller) and of exactly how to interpret the “other” facility findings.

Not all signal function indicators suggested by past literature were available in the SPA data, therefore, we were unable to exactly replicate the scoring system used by other authors, and cannot directly compare our findings to those of other papers. This is especially true for the EmNC indicators, for which we were unable to create any score. Additionally, some indicators were measured differently in the two surveys, reducing our ability to make an exact comparison between the two countries.

Our measures of patients’ experiences were also limited, and we do not know if these measures are actually representative of people’s experiences or if other types of questions would better capture experiential quality. More research is needed to see if these measures of ANC quality are the appropriate indicators to best understand what care women receive during their pregnancies. For example, in-depth interviews and longer and more detailed surveys have been used in other studies to assess the quality of the patient’s experiences of ANC or delivery care [24, 25].

Despite these limitations, this study builds upon current literature in a number of ways. It applies existing measurement tools of quality of facilities from Gabrysch et al. [10] and Nesbitt et al. [6] to two new locations – Kenya and Namibia and does so using a representative sample of all facilities in each country. This study allows for cross-country comparison using datasets that are both large and nationally representative (Nesbitt et al. included 86 facilities in their sample, while SPA sampled 403 in Kenya and 256 in Namibia). The data used in this analysis was collected using random sampling, so the findings should be more generalizable to the countries of interest as a whole. The fact that the status of obstetric and newborn emergency care in this larger and random sample of facilities were generally similar to the findings in Ghana add support to the robustness to those findings. Additionally, this study expands upon others by looking also at indicators of quality of care for ANC, and comparing the quality of ANC care to patient perceptions of their experiences.

In summary, a large proportion of facilities are lacking in many basic and emergency essential services, and few have all of the recommended services to improve maternal and neonatal health outcomes. These findings reflect past literature which found severe deficiencies in women’s reports of components of care received in antenatal and delivery care services in sub-Saharan Africa [26, 27]. The findings of this analysis both responds to, and underscores the importance of the call made by Graham and Varghese to include user perspectives while assessing quality of care in order to understand the full picture of the continuum of care [5]. However, we find few associations between perceived and actual care received. This could be because women’s perceptions of care are based in other types of quality, such as interpersonal interactions or other physical factors such as the physical attractiveness or cleanliness of the facility.

## Conclusions

More and more women globally are delivering in facilities. However, the quality of care and services available to these women when they reach a facility is not uniform, nor is it close to reaching the level of care recommended by WHO and other standard-setting agencies. It is essential that we turn our attention to improving the services available to women at facilities, and in this context to understanding more clearly what influences women’s perceptions of the quality of care and how these perceptions, often at odds with clinical quality, influence health seeking decisions. High and growing rates of facility deliveries present an opportunity to improve maternal and neonatal survival. Assuring quality within facilities and assuring that usage is associated with quality should be priorities in order to make use of this opportunity.
